# Chinese Food Image Database for Eating and Appetite Studies

**DOI:** 10.3390/nu14142916

**Published:** 2022-07-15

**Authors:** Xinhang Li, Yue Pan, Yan Han, Qianlin Liang, Xinmeng Yang, Xia Meng, Xiao Gao

**Affiliations:** 1 Faculty of Psychology, Southwest University, Chongqing 400715, China; leexinhang0209@gmail.com (X.L.); 18633990725@163.com (Y.P.); hanyan6766@163.com (Y.H.); liangql0626@163.com (Q.L.); xinmeng.yang@wur.nl (X.Y.); mx1520985371@163.com (X.M.); 2 Division of Human Nutrition and Health, Wageningen University and Research, Stippeneng 4, 6708 WE Wageningen, The Netherlands; 3 Key Laboratory of Cognition and Personality (Ministry of Education), Southwest University, Chongqing 400715, China

**Keywords:** food image, database, dietary decision-making, food cues, obesity

## Abstract

Modern people live in an environment with ubiquitous food cues, including food advertisements, videos, and smells. Do these food cues change people’s eating behavior? Since diet plays a crucial role in maintaining health, it has been researched for decades. As convenient alternatives for real food, food images are widely used in diet research. To date, researchers from Germany, Spain, and other countries have established food photo databases; however, these food pictures are not completely suitable for Chinese studies because of the ingredients and characteristics of Chinese food. The main goal of this research is to create a library of Chinese food images and to provide as complete a data reference as possible for future studies that use food images as experimental material. After standardized processing, we selected 508 common Chinese food pictures with high familiarity and recognizability and attached detailed classifications concerning taste, macronutrients, calories, and participants’ emotional responses to the pictures. Additionally, with food pictures as material, we conducted research on how people make dietary decisions in order to identify the variables that may affect a person’s food choices. The effects of individual perceived healthiness and palatability, gender, BMI, family income, and levels of emotional and restricted eating were examined using eating decisions based on healthiness and palatability as dependent variables. The results showed that people with low household incomes are more likely to be influenced by food taste in their dietary decision-making process, while individuals with high household incomes are more likely to consider the healthy aspects of food. Moreover, parental BMI affects what children consume, with children who have parents with higher BMIs being more prone to overlook the healthiness value of food.

## 1. Introduction

Obesity is one of the most highlighted health topics in the modern world. Currently, there are more than 2 billion overweight or obese people worldwide, and the rate of overweight and obesity continues to rise [1]. In 2016, a report by *The Lancet* showed that China’s obese population ranks first in the world [2]. Factors such as genetics, diet, eating behavior, culture, and environment can increase the proportion of the obese and overweight population to different degrees [3,4]. Studies show that obesity is caused by multiple interactions between genes and environmental factors [5]. However, the origin of obesity is excessive energy intake, especially excessive intake of sugar, salt, and fat [6].

Food cues in the environment are one of the noticeable reasons for excessive food intake, thereby inducing increased obesity rates. People are exposed to a great variety of environmental food cues, especially those related to/showing high-calorie food, such as barbecue, hamburgers, pizza, and fried chicken. Advertisers try to make food pictures more attractive to persuade people to consume more. Furthermore, these attractive and high-energy foods are usually convenient and affordable, which also motivates people to buy them [7]. When it comes to food affordability and accessibility, it is crucial to consider the social dominants that drive inequalities in healthy eating and cover a wide range of factors from the individual to society, such as daily living, education, and work, as well as policies and markets. People are inevitably divided into social, political, economic, and cultural groups as a result of these circumstances, resulting in societal stratification. Food affordability and accessibility for various groups naturally vary, which has an impact on their dietary preferences since it is difficult for individuals to pick food that is out of their reach, and finally leads to disparities in quality of life [8]. Corresponding to the influencing factors, researchers have explored some effective intervention methods through many empirical studies; nudge strategies are one of them, influencing individuals’ dietary decisions by cues without awareness and high cognitive effort. The exploration in the intervention field has also led to research focusing on the factors of individual dietary behavior, thereby promoting the improvement of the populations’ diet [9].

The impacts of environmental factors on food intake have been studied in many different fields, including psychology, neuroscience, and nutritional science. The environment influences individuals’ eating behavior by influencing cognitive, emotional, or external sensory cues, which in turn affect the so-called transient food intake system [10]. In this way, long-term exposure to a wide range of high-energy delicious foods, food advertisements, and increasingly competitive work environments can change people’s eating behaviors, most commonly in the form of increasing obesity or overeating [11]. Overt food images deeply influence people’s eating behavior. Therefore, visual food cues can be regarded as conditioned stimuli that are associated with the hedonic and homeostatic effects of ingestion [12].

Obese individuals tend to be attracted by food and eating stimuli, that is, they show an attentional bias to such stimuli. Yokum and Stice suggested that overweight and obesity are associated with attentional biases to food cues [13]. Individuals who respond strongly to reward circuits in response to food cues have a higher risk of weight gain. Obese individuals have higher behavioral tendencies to select high-energy food and are slower at avoiding particularly high-calorie snack food [14]. Additionally, compared with normal-weight individuals, the obese were more likely to orient to food images and had longer fixation times, even when they were full [11,15].

Neuroimaging studies corroborate these findings. Viewing food images during starvation may induce activation in the prefrontal cortex, the orbitofrontal cortex, and the amygdala. Changes in the calories in a food image can change food motivation, as high-calorie food images induce activation in the medial and lateral prefrontal cortex, and low-calorie food images induce activation in the orbitofrontal cortex [16,17]. In conclusion, current studies of food visual cues and neural mechanisms mainly show the following findings: brain activation on the edge of the reward area and system caused by food visual cues differs between obese individuals and healthy weight people, and, especially when viewing high-calorie food visual cues, the activation level in these brain regions of obese and overweight people is higher than that of healthy individuals [18,19].

### 1.1. The Establishment and Studies of Existing Food Image Databases

Food images, as a kind of visual food cues, can be used as effective and convenient substitutes in studies of eating behaviors in different fields. However, the food images used in most studies lack standardized processing, are only involved in one study, and vary across labs. To improve the comparability and consistency of studies, some researchers and laboratories developed food picture databases. The following three existing food image databases were chosen as references based on how the images were obtained, the aspects of the information provided, and the emphasis of the research objectives.

Jens Blechert et al. established a food image database which provides 896 food images (continuously updated) and 315 non-food images as well as comparisons [20,21]. The researchers considered the following points when they built the food image library: (1) the food’s calorie content is one of the most important factors influencing eating behavior decisions and neural activities [22,23]; (2) macronutrients in the food are taken into consideration; and (3) the classification of the food. The researchers computed relevant image properties that characterize the images’ physical appearance using customized scripts written in MATLAB R2011b. In terms of food composition, the German photo library collects the contents of calories and macronutrients (fat, protein, and carbohydrates), and pictures are classified by food type, processing degree, energy content, and taste. One of the reasons for choosing this database as a reference is because it has more pictures accessible than other collections and it regularly adds new images. Moreover, the researchers have standardized the photographs and provided data including physical characteristics, nutrition, and categorization for later users—aspects that are similarly crucial to take into account while creating a food image library of China.

The food photo library established by Lisette Charbonnier et al. aims to build a high-quality standardized food image library for European researchers to study eating behavior [24]. To reduce the shortcomings of the German food image library, such as decreases in attractiveness, size deviation, and non-conformity in eating behavior, researchers took photographs in the laboratory and quantified the angle, light, and distance to ensure the consistency of all pictures in the collection environment. All images were processed by MeVisLab software and Elastix software to unify the image background. Furthermore, they proposed that it is important to make the food pictures more similar to real food so that attention is paid to recognizability, attractiveness, energy content, and healthiness (especially the comparison between high- and low-calorie foods). The evaluation of this photo library mainly focuses on four items, including recognizability, liking, perceived caloric content and healthiness. All food was dressed on the plate (with the same size plate as a reference) and assessed by adults from Scotland, the Netherlands, and Greece. There are a total of 80 pictures with a recognition degree above 85% in the assessments in these three countries (40 high-calorie and 40 low-calorie food images, containing salty and sweet tastes). This research’s focus was not only on the calorie content of the meal but also on the calories perceived by participants when seeing images of the food, which was one of the motives for our research. When studying the factors that influence eating decisions, we focus on the individual’s perception of how delicious and healthy the meal is, as this varies from person to person and also differs from the actual energy content of the food. As for image collection, except for small food products such as M&Ms, most of the food was chopped into uniformly small pieces, placed in the same containers, and photographed in a tightly controlled laboratory setting. Obviously, the pictures have been meticulously processed, but the state of the food in the images differs extremely from that in real life, which may lead to a lower ecological validity, thereby impeding the real-world application of the experimental results. That is why we did not choose this method of image capture when building the image library.

The Open Library of Affective Foods (OLAF) is another food image library containing 96 food images. There are two differences between OLAF and other food image libraries: (1) The first difference is the source of the pictures. This food image library collected the photo materials from scenes photographed in restaurants or at home when the food had just finished cooking, not from the Internet or recipes, trying to reproduce the daily eating scene as much as possible. On the other hand, there is a gap between the image quality (brightness, contrast, clarity) and other aspects of the images and other image libraries. As visual stimuli, the presence of unrelated distracting content (e.g., alcoholic beverages) in the food background may affect the research results. (2) The second difference is the comparison between food pictures and affective pictures. Participants need to assess the valence, arousal, dominance, and food craving for these pictures. Results show that food pictures were similar to neutral pictures in pleasure and arousal, and high-calorie foods received higher scores in affective ratings. Unlike the above two image libraries, researchers focused on the emotional dimensions of food visual cues, and one of the purposes of building this food picture library was to connect food images and affective pictures (based on the International Affective Picture System), allowing researchers to better understand how healthy individuals and patients with diet and weight-related problems or disorders process food emotionally [25]. This is one of the reasons why this image library was picked as a reference source. The Chinese Food Image Database also provides an understanding of the effects of food images on people in terms of motivational and emotional dimensions, providing more thorough referenceable data for studies such as determining whether there is an association between motivational level and emotional eating.

### 1.2. Research Significance

Currently, food pictures selected randomly from the Internet or cookbooks are mostly used as experimental materials in studies of eating behavior among Chinese samples, which lack quantitative data on image characteristics and food composition, and these pictures only used evaluations by small samples to conduct experimental research [26,27]. Although some researchers have built food picture libraries, these food images are not completely suitable for studies conducted in China, as Chinese food is mostly different from food from other countries, especially in cooking materials and methods. The recognition and familiarity of many food items in the above databases are relatively low for Chinese participants, which may have an adverse impact on the accuracy and validity of the results. This is the main reason why constructing a Chinese food image database is necessary and valuable.

Dietary decision-making is closely associated with health, and to a certain extent, is based on visual food cues. Food pictures, as a kind of food visual cue, provide great convenience to dietary decision-making research. Many factors contribute to dietary decision-making, such as BMI, age, and family [13,28].

### 1.3. Research Purpose and Hypothesis

In the present study, we first aimed to create a database of a Chinese food image library. Every food item in the database includes physical characteristics of the images (color, size, contrast, brightness, and complexity), macronutrients, and food category. The construction of the Chinese Food Image Database referenced research methods used in previous food databases. Second, we assessed these pictures through the following factors: affective ratings, arousal, liking and wanting. We also collected participants’ demographic and other information about dietary behavior and personality. Finally, this study mainly focuses on the influence of individual perceived food taste, health level, and other factors including the family income and parents’ BMI on their dietary decision-making.

The following two main hypotheses were formulated for the study results:

**Hypothesis** **1.** *Individuals with high family income are more likely to pay attention to the healthiness of food rather than the palatability*.

**Hypothesis** **2.** *The higher their parents’ BMI is, the fewer the individuals consider the health factors, and the easier it is for them to make dietary decisions based on the taste of food*.

## 2. Methods

### 2.1. The Collection and Processing of Food Images

#### 2.1.1. Materials

The source of the photographs is the Internet rather than photographs taken in the field. These photographs contain common Chinese foods and some common Western ones. The selection of the food was based on familiarity, integrity, and photo angle and clarity. The food (including containers) in the pictures is all presented on a white background, and the pixels are uniformly adjusted to be 650 × 400 (96 dpi, color mode is RGB).

#### 2.1.2. Image Physical Characteristics

We computed images with MATLAB script written by the diet behavior laboratory of Salzburg University (MATLAB R2011b, MathWorks, Inc., Natick, MA, USA). This script is used to analyze batch data of all images in the image library in terms of image size, color, brightness, internal contrast, and complexity of objects in the images [20].

#### 2.1.3. Macronutrients

We collected the calorie and nutrient composition of the food in the pictures, and data based on the Chinese Food Composition (second edition), Internet food nutrient composition databases (http://www.ars.usda.gov, accessed on 16 May 2016, www.inmu.mahidol.ac.th/thaifcd/home/php, accessed on 16 May 2016), and the composition information provided by the food package. The unit of energy is calories per 100 g, and the nutrients include protein, fat, carbohydrates, dietary fibre, cholesterol, fatty acids (saturated fatty acids, monounsaturated fatty acids, and polyunsaturated fatty acids, respectively), vitamin C, and vitamin E in grams per 100 g or milligrams per 100 g, respectively.

#### 2.1.4. Category

Based on the food characteristics, we classified the food images in four ways: (1) based on the variety of foods, we categorized the food images into ten types, including fruits, vegetables, staple foods, meat dishes, seafood, vegetarian dishes, nuts, dessert, and Chinese dessert; (2) based on the degree of processing, we categorized the food images into two groups, i.e., whole processed food (e.g., Chinese dishes, desserts) and non-processed food (e.g., vegetables and fruits); (3) based on caloric content and an assessment by two bromatology professors from Southwest University, we categorized the food images into two groups, i.e., low caloric and high caloric food groups; and (4) based on the taste, we categorized the food images into four groups, i.e., sweet, salty, spicy, and sour foods.

### 2.2. Participants

The sample consisted of 989 students from junior high school, senior high school, and university in China. All the pupils in two junior high school grades and one senior high school grade were chosen for the secondary education age group. The university group consisted of sophomores and juniors who had chosen to take one of the general psychology optional courses. Table 1 summarizes the participants’ demographic characteristics.

### 2.3. Demographics and Scales

The survey commenced with a collection of demographics (age, gender, nationality, etc.), eating habits, and information about the participants’ parents (including family income and BMI), which were self-reported by participants. After that, the entire experimental design is divided into two main parts, the filling of the scale and the rating of the food pictures. The Chinese version of the DEBQ (Diet Eating Behavior Questionnaire) was used in the study, which contains three scales: Restrained Eating (RS), Emotional Eating (Em), and External Eating (Ex) [29]. This scale has been cross-culturally validated in previous studies and has high reliability and validity. For the university student sample and the adolescent sample, the Cronbach’s alpha coefficients for the total scale and each subscale were above 0.85, similar in results to the Dutch sample, and had stable construct validity, indicating that the DEBQ scale is cross-culturally stable and has good applicability to the Chinese population [30,31]. The mean DEBQ scores are shown in Table 1.

### 2.4. Image Rating Procedure

The researchers divided 508 images into eight groups, balanced the number of different food types, and manipulated a pseudorandom order in each group. Each group contained 63 or 64 images. In the formal experiment, each group of participants only evaluated one group of pictures, that is, there were eight groups of participants at each educational stage (six groups of college students). Ultimately, each food image was rated by both adults (*Mean* = 53.8, *SD* = 19.5) and adolescents (*Mean* = 70.7, *SD* = 11.7, consisting of 32.9 (*SD* = 7.7) junior high school students and 38.4 (*SD* = 9.1) senior high school students).

The rating task consisted of three parts. To establish a Chinese food image library, it is necessary to ensure that the food in the pictures can be recognized and is familiar to the public. Thus, the first part was about the recognizability and familiarity of the picture. There were two items in total, both of which used two-point scales for ‘Yes’ and ‘No’ answers. If the subject’s judgment for the first item on the recognizability of the picture was ‘No’, then the subsequent evaluation of the picture was completed and the evaluation of the next picture proceeded directly.

**Figure 1:**
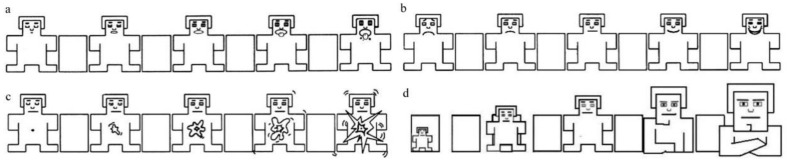
Self-Assessment Manikin (SAM): (**a**) wanting, (**b**) valance, (**c**) arousal, and (**d**) dominance.

The second part was about motivation/emotional experience when looking this food. University students used a 9-point Likert scale to evaluate the 5 items: liking, wanting, valance, arousal, and dominance. The questions were: ‘How do you like the food?’ (1, not at all–9, very much), ‘How much do you desire to eat the food?’ (1, no craving at all–9, strong craving), ‘How much pleasure do you feel when seeing this food?’ (1, very unhappy/sad–9, very happy), ‘How excited do you feel when seeing this food?’ (1, very calm–9, very excited/active), ‘How dominant do you feel when seeing this food?’ (1, out of control–9, very important/controllable). Middle school students used the graphical Self-Assessment Manikin Scale (SAM, see Figure 1) to assess wanting, pleasure, arousal, and dominance. Each SAM scale includes 5 dwarf patterns and 4 rectangular boxes, and subjects can be evaluated on 9 levels [25,32].

The third part was decision-making evaluation, including three items, which required participants to evaluate the healthiness and palatability of food shown in the pictures, and make dietary decisions based on these two factors. University students used a 9-point Likert scale, and middle school students used a 5-point Likert scale.

## 3. Results

### 3.1. Food Image Classification

**Figure 2:**
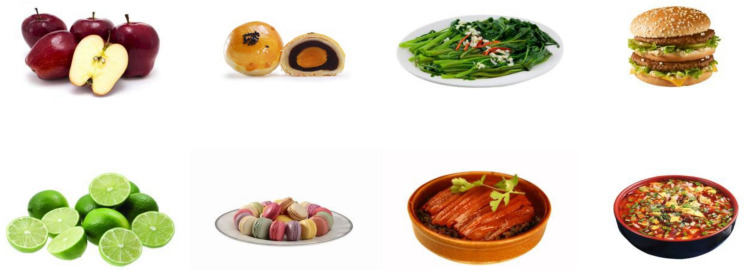
Samples of Chinese food images. The images are sourced from open-source photo websites.

After screening and analysis, the Chinese food image library established in this study contains 508 food images. According to the food pictured, the main types of images were divided into fruits (a total of 57 images, accounting for 11.2% of all food images), vegetables (12.4%), meat dishes (19.3%), seafood (12.0%), pulses (5.1%), staple foods (8.3%), Chinese pastries (9.6%), desserts (11.2%), nuts (5.0%), and other foods (5.9%). Furthermore, food pictures were divided into different categories according to the degree of food processing, energy content, and major tastes in the pictures. Of these, 120 pictures (23.6% of all pictures) were placed in the unprocessed food group and 388 pictures (76.4% of all pictures) were placed in the processed food group. There are 174 images in the low-energy food group (34.3% of all pictures) and 334 images in the high-energy food group (65.7% of all pictures). The sweet food group contains 167 images (32.9% of all pictures) and the salty delicious food group has 192 images (37.8% of all pictures). A total of 43 pictures (8.5%) were placed in the spicy food group, and 29 pictures (5.7%) were placed in the sour food group. The main tastes of a total of 77 food images (15.2% of all images) were difficult to determine. Samples of the Chinese food images are shown in Figure 2.

### 3.2. Identifiability and Familiarity Ratings

Overall, the average identifiability and familiarity of food images was 83.1% (*SD* = 17.6%) and 91.8% (*SD* = 6.8%), respectively. Participants in different education stages also differed in their image recognition and familiarity. In terms of identifiability, the mean recognition evaluation value of pictures by participants was 85.2% (*SD* = 15.8%) for junior high school students, 84.6% (*SD* = 20.2%) for high school students, and 79.1% (*SD* = 22.3%) for university students *(F* = 29.411, *p* < 0.001). In terms of familiarity, the average familiarity rating of the pictures in junior high school students was 94.0% (*SD* = 6.4%), that of high school students was 91.7% (*SD* = 9.5%), and that of university students was 89.7% (*SD* = 10.1%). The main effect of age (education stage) is significant (*F* = 5.675, *p* < 0.01).

### 3.3. Statistical Analysis

To explore the importance of healthiness and palatability in the participants’ dietary decisions, we took everyone’s ratings of ‘choose to eat’ as the dependent variable, and the ratings of healthiness and palatability of food pictures were taken as independent variables in the multiple stepwise regression analysis. The regression equation was established to obtain the standardized coefficient of each person’s rating of healthiness and palatability in the dietary decision. The results show that the mean *β* of palatability was significantly greater than that of healthiness in the participants’ dietary decision-making, and the specific coefficient is shown in Table 2.

Regarding gender, there was no significant difference between males and females in the influence of healthiness on dietary decision-making, while females considered palatability more than males. For different ages, the influence of healthiness and palatability on university students’ dietary decision-making was significantly greater than that of high school students. However, there was no significant difference in the mean *β* of healthiness and palatability among participants with different BMI scores. The specific values are shown in Table 2.

We also analyzed the correlation between the DEBQ score and the standardized coefficients of healthiness and palatability. The result is shown in Table 3. The DEBQ-RS score was related to the coefficient of healthiness, while the DEBQ-Ex score was related to the coefficient of palatability.

Family income also played a role in participants’ dietary decision-making. As shown in Table 4, the coefficient of healthiness is relevant to family income and parents’ BMI. The higher the family income is, the more health factor is valued in dietary decisions. The higher the parents’ BMI is, the fewer health factor is valued in the children’s dietary decisions.

According to the scores of all participants, the average palatability and healthiness ratings were considered independent variables, while the choose to eat ratings were the dependent variable in regression analysis; high-calorie and low-calorie food coefficients were calculated separately. Then, an independent sample t-test was used to analyze the difference between low-calorie and high-calorie foods. Table 5 shows that, compared with dietary decision-making in the high-calorie food pictures, participants paid more attention to the palatability of low-calorie food. By contrast, participants considered the healthiness ratings of high-calorie food more than those of low-calorie food.

## 4. Discussion

The establishment of the database mainly considered image features such as physical characteristics, nutritional composition, classification, identification, and familiarity. The method referred to the establishment of existing food picture databases and methods of picture analysis and adjusted them according to food pictures that conform to the dietary habits of Chinese people. Considering the number of images and the image quality, the pictures were mainly derived from the Internet. Although images taken in real life have higher ecological validity, the background of the picture is less controllable and background interference may affect the use of the images. For example, in the photo library established by the OLAF laboratory, in addition to specific food, some images show alcoholic beverages in the background, which may change the individual’s perception of the food itself. Therefore, the food in our image database is presented on a white background, and the image size, color, brightness, contrast, and other physical characteristics of the image were adjusted.

In terms of image classification, the Chinese Food Image Database integrates the classification standards of food in the existing photo libraries, and classifies food images in terms of processing degree, taste, and energy. Additionally, the database also provides more detailed data on the content of food ingredients, so that users can re-classify or screen the pictures according to different experimental needs and research purposes, instead of being limited by the existing classification. Since the individual’s familiarity with the food presented in the pictures and the recognizable performance of the food can directly affect the use effect of the food image database and affect subjects’ feelings and cognitions to a certain extent, the research focuses on the two aspects of the pictures’ recognizability and familiarity. The results show that the familiarity and recognizability of the pictures are relatively high in participants across the three education stages, which confirms the universality and recognizability of the selected materials.

Some research has found that a food’s tastefulness is related to an individual’s love for it. This leads to the idea that taste represents a food’s hedonic value, and healthiness represents its nutritional value [33,34]. According to our results, people attach more importance to the taste of food when making dietary decisions, and to healthiness as they get older. Compared with men, women pay more attention to the deliciousness of the food, which is consistent with previous studies [35,36]. What we did not expect, however, was that there was no significant difference in the effects of deliciousness and healthiness in diet decision making between individuals with different BMI. The reason may be that participants with high BMI may pay more attention to rejecting high caloric food or their attention is distracted from these food cues [15,37].

Family income and parents’ BMI also had a great influence on dietary decisions. The higher the family income is, the higher the coefficient of healthiness is. Those who have better economic background will consider healthiness factors more important when making dietary decisions, which is consistent with our study hypothesis. There are several possible explanations for this result. As mentioned at first, high-energy food is always cheap and easy to buy, so, with higher incomes, families may have more disposable money to choose and consume organic healthy food. Low income often implies a low educational experience, unstable work, and relatively poor living conditions, and this group tends to be more likely to experience food insecurity, unhealthiness, and higher levels of diet-related illnesses [8]. It is also worth mentioning that parents’ BMI is negatively correlated with the weight of food health factors in children’s decision-making process. Meanwhile, overweight parents may instill in their children a habit of unconsciously ignoring the healthiness factors of food when eating [38]. It is undeniable that the home environment plays a crucial role in shaping the dietary behaviors of families, and the impact of the home food environment on individuals’ weight has received attention in studies in recent years. High-calorie food in a home is related to high energy intake, and the accessibility of fruit and vegetables is associated with low energy intake [39,40]. Compared with normal-weight adults, overweight adults have fewer low-fat snacks, fruit and vegetables and more high-fat snacks in the home. So, inevitably, they also purchase more high-fat and high-calorie food, and this shopping habit may influence their children’s eating behavior [41]. Many studies have shown that interventions (such as raising taxes on unhealthy meals while subsidizing healthy fruits and vegetables, including meal planning in the school curriculum, giving free or subsidized fruits and vegetables, and eliminating unhealthy food from school canteen menus and vending machines) can reduce inequities in healthy eating caused by income and family environment in the short term, but previous researchers have proposed that what these interventions may overlook is the potentially uneven distribution of factors that promote healthy eating opportunities, which could backfire by not only failing to reduce inequities but also widening the gap in healthy eating [8].

DEBQ-RS score was significantly correlated with the coefficient of healthiness, indicating that compared with those participants who do not restrict their diet, those who are on a restrictive diet will consider healthiness factors more when making dietary decisions. The DEBQ-Ex score was significantly correlated with the coefficient of palatability. When participants eat out with others or go on an exogenous diet, they consider deliciousness more than health [42].

The energy of food is one of the factors affecting dietary decisions. Compared with low-calorie food, seeing high-calorie food makes people value healthiness over palatability, while people emphasize the taste of low-calorie food. Simply put, when people choose between delicious, high-calorie options, such as eating two identical cakes, they may prefer the one with an extra cherry on top.

This study also had certain limitations, which can be circumvented in future studies. Firstly, although relevant data provided food pictures in as much detail as possible, some information about food energy and the component content is still missing. Because data about the nutritional content and energy of food in units of 100 g came from the available network resources, it was difficult to calculate the total nutrient reference value of food in pictures. Moreover, some pictures of processed Chinese dishes failed to obtain accurate information on ingredients and energy, so the classification of high and low energy only depended on the classification results of experts from the college of food science. Secondly, despite a large number of participants, all of them were students, and the narrow age range of the participants may have affected the results of the experiment. In following research, we will use the image library in experiments for all age groups and improve the reference data of the database. Moreover, this experiment was carried out in the southwest of China, a region with a vast territory and various food cultures. Although local delicacies have been continuously circulated across the country in recent years, the dietary habits of residents in different regions are still quite different. Therefore, this is also an interference factor that may have a certain impact on the results. Future research should take regional factors into account and balance regional differences as much as possible. In this study, we mainly focused on the influence of family income and parents’ BMI on dietary decision-making. However, many factors can affect dietary behavior, such as dining in school and who is responsible for the decision-making of meals in the family. 

## 5. Conclusions

We attempted to investigate factors that influence dietary decisions in this study, but we did not further explore or validate the effect of visual food stimulus cues in intervening in individuals’ eating decisions. Therefore, future studies may use food pictures as material and nudge strategies to try to change individuals’ eating decisions and subsequent eating behaviors.

## Figures and Tables

**Table 1:** Demographic characteristics of the sample.

Participants’ Characteristics	*n* *(%)*	*Mean (SD)*
Gender	989	
Female	666 (67.34%)	-
Male	323 (32.66%)	-
		
Age Group	989	
Junior high school	263 (26.59%)	12.84 (0.79)
Senior high school	307 (31.04%)	15.89 (0.80)
University	419 (42.37%)	19.95 (0.94)
		
Body Mass Index	952	
BMI ≤ 18	283 (29.73%)	17.17 (1.01)
18 < BMI ≤ 24	586 (61.55%)	20.73 (1.44)
BMI > 24	83 (8.72%)	25.99 (1.78)
		
DEBQ-RS	793	22.40 (8.13)
DEBQ-Em	792	28.84 (12.50)
DEBQ-Ex	811	30.74 (8.58)

Note: DEBQ-RS = Dutch Eating Behavior Questionnaire-Restrained Eating subscale; DEBQ-Em = Dutch Eating Behavior Questionnaire-Emotional Eating subscale; DEBQ-Ex = Dutch Eating Behavior Questionnaire-External Eating subscale.

**Table 2:** The mean *β* of healthiness and palatability in the participants’ choice to eat.

	Healthiness		Palatability	
Characteristics	*Mean*	*SD*	*Mean*	*SD*
Total	0.2508	0.2620	0.6277	0.2698
				
Male	0.2373	0.2383	0.5958 **	0.2622
Female	0.2574	0.2727	0.6431 **	0.2723
				
High school	0.1996 ***	0.2380	0.6114 **	0.2679
University	0.3203 ***	0.2771	0.6498 **	0.2713
				
BMI ≤ 18	0.2264	0.2495	0.6346	0.2592
18 < BMI ≤ 24	0.2615	0.2636	0.6239	0.2755
BMI > 24	0.2655	0.2764	0.6306	0.2697

*** *p* < 0.001, ** *p* < 0.01.

**Table 3:** The results of correlation analysis between DEBQ and choice to eat.

	Healthiness (*β*)	Palatability (*β*)	DEBQ-RS	DEBQ-Em
Healthiness (*β*)	1.000			
Palatability (*β*)	−0.684 **	1.000		
DEBQ-RS	0.150 **	−0.053	1.000	
DEBQ-Em	0.034	0.029	0.267 **	1.000
DEBQ-Ex	−0.015	0.150 **	0.249 **	0.440 **

DEBQ-RS = Dutch Eating Behavior Questionnaire-Restrained Eating subscale; DEBQ-Em = Dutch Eating Behavior Questionnaire-Emotional Eating subscale; DEBQ-Ex = Dutch Eating Behavior Questionnaire-External Eating subscale. ** *p* < 0.01.

**Table 4:** The result of correlation analysis between family income and choice to eat.

	Healthiness (*β*)	Palatability (*β*)	Family Income	Father’s BMI
Healthiness (*β*)	1.000			
Palatability (*β*)	−0.684 **	1.000		
Family income	0.073 *	0.050	1.000	
Father’s BMI	−0.069 *	0.014	−0.001	1.000
Mother’s BMI	−0.080 *	−0.004	−0.014	0.813 **

** *p* < 0.01, * *p* < 0.05.

**Table 5:** Image-based eating decisions (*β*).

		Standard Beta		*t*	*p*
Total	Palatability	0.851		79.868	<0.001
	Healthiness	0.247		23.215	<0.001
					
Low-calorie food	Palatability	0.860		52.835	<0.001
	Healthiness	0.196		12.062	<0.001
					
High-calorie food	Palatability	0.837		52.9410	<0.001
	Healthiness	0.233		14.7720	<0.001

## Data Availability

The data are not publicly available due to privacy and ethical restrictions. The data presented in this study may be available conditionally from the corresponding author.
